# Retraction Note: Eye tracking: empirical foundations for a minimal reporting guideline

**DOI:** 10.3758/s13428-023-02285-0

**Published:** 2023-11-16

**Authors:** Kenneth Holmqvist, Saga Lee Örbom, Ignace T. C. Hooge, Diederick C. Niehorster, Robert G. Alexander, Richard Andersson, Jeroen S. Benjamins, Pieter Blignaut, Anne-Marie Brouwer, Lewis L. Chuang, Kirsten A. Dalrymple, Denis Drieghe, Matt J. Dunn, Ulrich Ettinger, Susann Fiedler, Tom Foulsham, Jos N. van der Geest, Dan Witzner Hansen, Samuel B. Hutton, Enkelejda Kasneci, Alan Kingstone, Paul C. Knox, Ellen M. Kok, Helena Lee, Joy Yeonjoo Lee, Jukka M. Leppänen, Stephen Macknik, Päivi Majaranta, Susana Martinez-Conde, Antje Nuthmann, Marcus Nyström, Jacob L. Orquin, Jorge Otero-Millan, Soon Young Park, Stanislav Popelka, Frank Proudlock, Frank Renkewitz, Austin Roorda, Michael Schulte-Mecklenbeck, Bonita Sharif, Frederick Shic, Mark Shovman, Mervyn G. Thomas, Ward Venrooij, Raimondas Zemblys, Roy S. Hessels

**Affiliations:** 1 Department of Psychology, Nicolaus Copernicus University, Torun, Poland; 2 Department of Computer Science and Informatics, University of the Free State, Bloemfontein, South Africa; 3 Department of Psychology, Regensburg University, Regensburg, Germany; 4 Experimental Psychology, Helmholtz Institute, Utrecht University, Utrecht, The Netherlands; 5 Lund University Humanities Lab and Department of Psychology, Lund University, Lund, Sweden; 6 Department of Ophthalmology, SUNY Downstate Health Sciences University, Brooklyn, NY USA; 7 Tobii Pro AB, Danderyd, Sweden; 8 Social, Health and Organizational Psychology, Utrecht University, Utrecht, The Netherlands; 9 TNO, Soesterberg, The Netherlands; 10 Department of Ergonomics, Leibniz Institute for Working Environments and Human Factors, Dortmund, Germany; 11 Institute of Informatics, LMU Munich, Munich, Germany; 12 Institute of Child Development, University of Minnesota, Minneapolis, USA; 13 School of Psychology, University of Southampton, Southampton, UK; 14 School of Optometry and Vision Sciences, Cardiff University, Cardiff, UK; 15 Department of Psychology, University of Bonn, Bonn, Germany; 16 Vienna University of Economics and Business, Vienna, Austria; 17 Department of Psychology, University of Essex, Essex, UK; 18 Department of Neuroscience, Erasmus MC, Rotterdam, The Netherlands; 19 Machine Learning Group, Department of Computer Science, IT University of Copenhagen, Copenhagen, Denmark; 20 SR Research Ltd, Ottawa, Canada; 21 Human-Computer Interaction, University of Tübingen, Tübingen, Germany; 22 University of British Columbia, Columbia, Canada; 23 Department of Eye and Vision Science, Institute of Life Course and Medical Sciences, University of Liverpool, Liverpool, UK; 24 Department of Education and Pedagogy, Division Education, Faculty of Social and Behavioral Sciences, Utrecht University, Utrecht, The Netherlands; 25 Department of Online Learning and Instruction, Faculty of Educational Sciences, Open University of the Netherlands, Heerlen, The Netherlands; 26 University of Southampton, Southampton, UK; 27 School of Health Professions Education, Faculty of Health, Medicine, and Life Sciences, Maastricht University, Maastricht, The Netherlands; 28 Department of Psychology and Speed-Language Pathology, University of Turku, Turku, Finland; 29 TAUCHI Research Center, Computing Sciences, Faculty of Information Technology and Communication Sciences, Tampere University, Tampere, Finland; 30 Institute of Psychology, University of Kiel, Kiel, Germany; 31 Lund University Humanities Lab, Lund University, Lund, Sweden; 32 Department of Management, Aarhus University, Aarhus, Denmark; 33 Center for Research in Marketing and Consumer Psychology, Reykjavik University, Reykjavik, Iceland; 34 Herbert Wertheim School of Optometry and Vision Science, University of California, Berkeley, CA USA; 35 Comparative Cognition, Messerli Research Institute, University of Veterinary Medicine Vienna, Medical University of Vienna, Vienna, Austria; 36 Department of Geoinformatics, Palacký University Olomouc, Olomouc, Czech Republic; 37 The University of Leicester Ulverscroft Eye Unit, Department of Neuroscience, Psychology and Behaviour, University of Leicester, Leicester, UK; 38 Department of Psychology, University of Erfurt, Erfurt, Germany; 39 University of Bern, Bern, Switzerland; 40 Max Planck Institute for Human Development, Berlin, Germany; 41 School of Computing, University of Nebraska-Lincoln, Lincoln, Nebraska USA; 42 Center for Child Health, Behavior and Development, Seattle Children’s Research Institute, Seattle, WA USA; 43 Department of General Pediatrics, University of Washington School of Medicine, Seattle, WA USA; 44 Eyeviation Systems, Herzliya, Israel; 45 Department of Industrial Design, Bezalel Academy of Arts and Design, Jerusalem, Israel; 46 Electrical Engineering, Mathematics and Computer Science (EEMCS), University of Twente, Enschede, The Netherlands; 47 Smart Eye AB, Göteborg, Sweden

**Retraction Note: Behavior Research Methods (2022) 55:364-416**

**https://doi.org/10.3758/s13428-021-01762-8**

The authors have retracted this article because a number of statements are supported by two references, Holmqvist (2015) and Holmqvist (2016), which should not have been used. Saga Lee Örbom, Ignace T. C. Hooge, Diederick C. Niehorster, Robert G. Alexander, Richard Andersson, Jeroen S. Benjamins, Pieter Blignaut, Anne-Marie Brouwer, Lewis L. Chuang, Kirsten A. Dalrymple, Denis Drieghe, Matt J. Dunn, Ulrich Ettinger, Susann Fiedler, Tom Foulsham, Jos N. van der Geest, Dan Witzner Hansen, Samuel B. Hutton, Enkelejda Kasneci, Alan Kingstone, Paul C. Knox, Ellen M. Kok, Helena Lee, Joy Yeonjoo Lee, Jukka M. Leppänen, Stephen Macknik, Päivi Majaranta, Susana Martinez-Conde, Antje Nuthmann, Marcus Nyström, Jacob L. Orquin, Jorge Otero-Millan, Soon Young Park, Stanislav Popelka, Frank Proudlock, Frank Renkewitz, Austin Roorda, Michael Schulte-Mecklenbeck, Bonita Sharif, Frederick Shic, Mark Shovman, Mervyn G. Thomas, Ward Venrooij, Raimondas Zemblys and Roy S. Hessels agree with this retraction. Kenneth Holmqvist is deceased.

